# Burn injury differentially alters whole-blood and organ glutathione synthesis rates: An experimental model

**DOI:** 10.4103/2321-3868.118934

**Published:** 2013-09-18

**Authors:** Zhe-Wei Fei, Vernon R. Young, Xiao-Ming Lu, Andrew B. Rhodes, Ronald G. Tompkins, Alan J. Fischman, Yong-Ming Yu

**Affiliations:** 1 Shriners Burns Hospital and Burn & Trauma Service, Massachusetts General Hospital, Harvard Medical School, Boston, MA, 02114 USA; 2 Laboratory of Human Nutrition, Massachusetts Institute of Technology, Cambridge, MA, 02142 USA

**Keywords:** Burn injury, glutathione, glutathione concentration, synthesis rate, whole blood

## Abstract

Previous studies from our laboratories revealed a reduced rate of whole-blood (WB) glutathione (GSH) synthesis in severely burned patients. To determine whether WB GSH metabolism is an indicator of the status of GSH metabolism in one or more of the major organs, we used a burn rabbit model to determine GSH concentrations and rates of synthesis in WB, liver, lungs, kidney, and skeletal muscle. L-[1-^13^C]-cysteine was infused intravenously for 6 h in rabbits at 3 days post-burn and in sham burn controls. WB and organ ^13^C-enrichment of cysteine and GSH was determined by gas chromatography/mass spectrometry. Plasma cysteine metabolic flux was increased significantly (*P* < 0.01) following burn injury. WB, liver, and lung GSH concentrations (*P* = 0.054, *P* < 0.05, and *P* < 0.05, respectively) and fractional rates of GSH synthesis (*P* < 0.05, *P* < 0.01, and *P* < 0.05, respectively) were reduced at 3 days post-burn. Kidney was unaffected. There also appears to be an increased rate of GSH transport out of the liver after burn injury. Hence, there is a differential impact of burn injury on tissue and organ GSH status, with WB qualitatively reflecting the changes in lung and liver. It will be important to determine whether these changes are due to alterations in the intrinsic capacity for GSH synthesis and/or availability of amino acid precursors of GSH.

## Introduction

The tripeptide glutathione (g-glutamyl-cysteinyl-glycine; GSH), present in millimolar quantities in various tissues, plays a critical role in the detoxification of the reactive electrophilic intermediates of oxidative metabolism and nitric oxide metabolism[1,2] and in redox-dependent cell signaling by modulating the oxidation state of critical protein cysteine residues.[3,4] More recent studies revealed that the GSH status is directly related to the outcome of ICU patients,[5,6] patients with immune deficiency[7,8] and other diseases.[9–13] GSH status also contributes to the worsening of the disease process.[14] Burn injury[15] and other critical illness[16] decrease the concentration of GSH in organs and intracellular organelles.[17,18] In burn patients, we reported a reduced rate of whole-blood GSH synthesis;[19] however, there is no information with which we might be able to judge how this latter change in burn patients might reflect that occurring in major organs. For investigating possible nutritional factors that might modulate GSH homeostasis under stressful conditions, we used an established rabbit model in the present study[20] to examine the effects of burn injury on the concentration and synthesis rates of reduced GSH in whole blood and major organs. Stable isotope tracer method was used to evaluate the GSH synthesis in addition to concentration changes *in vivo*. l-[^13^C]-cysteine was used as a tracer to label GSH in vivo, and a new gas chromatography/mass spectrometric method was developed for determination of the ^13^C-enrichment in intact GSH.[21]

**Table:** 

Access this article online	
**Quick Response Code:**	**Website:** www.burnstrauma.com
	**DOI:** 10.4103/2321-3868.118934

## Materials and methods

### Animal care and induction of burn injury

Thirteen male New Zealand white rabbits of body weight 2.5-3.0 kg purchased from Millbrook Breeding Labs (Amherst, MA) were used in this study. The rabbits were housed in the animal farm of the Massachusetts General Hospital with a 12-h light/dark cycle and fed with regular rabbit chow (Prolab Hi-Fiber Rabbit, 5P25; PMI Nutrition International, Inc., Brentwood, MO) and water *ad libitum*. The composition of the rabbit chow is shown in Table 1. During the first 3 days, the animals were acclimatized to the restraint cage and the custom-designed face mask for collection of expired air during the tracer study. On the fourth day, each rabbit was anesthetized by intramuscular injection of a mixture containing xylazine (5 mg/kg), ketamine (30 mg/kg), and acepromazine (1 mg/kg). The polyethylene catheters (PE 90 catheter with internal diameter of 0.034 inch and expernal diameter of 0.050 inch (Clay Adames Co. Parsippany, NJ)) attached with 3-cm silastic tips (ID, 0.030 in.; OD, 0.065 in.; Don Corning Co., Midland, NC) were implanted into the right carotid artery and external jugular vein, using aseptic procedures. The catheters were externalized via a subcutaneous tunnel at the interscapular area and occluded by intermittent injection caps (JELCO™; Critikon Inc., Tampa, FL) for later intravenous infusion and arterial blood sampling.

**Table 1:** Composition of the rabbit diet (Prolab® Hi-Fiber Rabbit 5P25*)

Ingredient	Content per 100 g of the diet†
Protein	16.5
Amino acids	
Arginine	0.79
Cystine	0.19
Glycine	0.52
Histidine	0.48
Isoleucine	0.82
Leucine	1.12
Lysine	0.98
Methionine	0.18
Phenylalanine	0.95
Tyrosine	0.77
Threonine	0.61
Tryptophan	0.25
Valine	1.07
Serine	0.87
Aspartic acid	1.93
Glutamic acid	3.21
Alanine	0.77
Proline	1.17
Fat	5.0
Fiber	21.9
Minerals	8.6
Total energy (kcal)	209

*Information provided by PMI Nutrition International, Inc., PO Box 19798, Brentwood, MO 63144

^†^The unit is g, except being specified

In one group, six rabbits were again anesthetized on day 5, as described above. The hair of the dorsum was shaved and then a 25% total body surface area (TBSA) third-degree burn was induced. The total surface area of the rabbit was determined as previously described[20]; we estimated that a full-thickness burn surface area of about 500 cm^2^ approximates to a 25% TBSA burn for rabbits weighing 2.5–3.0 kg. Therefore, an oval-shaped mark was made on the back of the rabbits along a computer-generated template with total area of 500 cm^2^. Then, with additional halothane inhalation to deepen the anesthesia, the back of the animal was immersed into boiling water along the mark for 10 s. A full-thickness burn was later verified by histology examination. The animal was allowed to recover from the anesthesia with oxygen inhalation. Resuscitation was conducted immediately with a 4-h intravenous infusion of saline (3 ml/kg, %TBSA). The burned area was wrapped with a sterile gauze bandage (Kerlix Lite®; Kendall Healthcare Products Co., Mansfield, MA) for protection. Another seven animals served as the control group. These were treated in the same manner as described for the burned animals, except that they received a sham burn injury by immersion in water at 37°C. The study protocol was approved by the Subcommittee on Research Animal Care of the Massachusetts General Hospital.

### Tracer infusion studies

**Figure 1:**
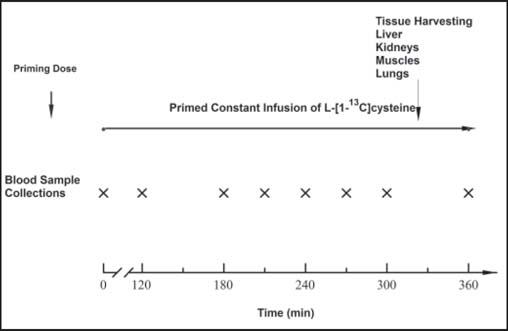
Protocol followed for tracer infusion studies to determine glutathione synthesis rates.

The stable isotope studies were conducted on the third day after induction of the thermal injury or sham burn when the animal was in hypermetabolic and protein catabolic state. Food was removed from the cage at 18:00 h on the day before the tracer study and the tracer infusion study started between 07:00 h and 08:00 h on the following day. Before starting the infusion, a blood sample (3 ml) was taken for evaluation of baseline ^13^C-enrichment of cysteine and GSH. Then, a primed constant infusion of [1-^13^C]-cysteine tracer (99% abundance; MassTrace, Inc., Woburn, MA) was given for 6 h. The targeted infusion rate was about 1 μmol/kg/min for the sham burn group and 0.85 μmol/kg/min for the burn group; tracer was delivered by an accurate pump (Model 920, Harvard Apparatus, Natick, MA), with a priming dose equivalent to a 60 min infusion dose, given by an intravenous bolus. Blood samples, 2.5 ml each, were drawn from the right carotid artery into 5 ml ethylenediaminetetraacetic acid (EDTA)-coated vacuum tubes (Vacutainer®, K_3_EDTA; Becton-Dickinson and Co., Franklin Lakes, NJ) at baseline and then at 60, 120, 180, 240, 270, 300, and 360 min after beginning the infusion. Aliquots of blood samples, 50 μl, were immediately mixed with 1 ml of ice-cold dithiothreitol (DTT, 20 mM in 1 M acetic acid) and frozen at −70°C. The plasma was separated immediately from the whole blood by centrifugation at −4°C. At the end of the infusion study, euthanasia was performed by intravenous injection of an overdose (>100 mg/kg) of sodium phenobarbital (Anpro Pharmaceutical, Arcadia, CA). The liver, lungs, kidney, and skeletal muscle (left quadriceps femoris) samples were taken immediately and frozen in liquid nitrogen. They were kept in a −70°C deep freezer until used for analysis (within 30 days). A schematic detailing the tracer study protocol is presented in Figure 1.

### Analytical methods

The procedure for analysis of whole-blood GSH concentration and isotopic enrichment has been described.[21] Briefly, the GSH derivative was monitored under negative chemical ionization conditions by selective ion monitoring (SIM) at nominal masses from *m/z* 477.1 to 480.1, corresponding to the most abundant and preponderant near-parent ion. The concentration of whole-blood GSH was determined, using synthetic [1,2-^13^C_2_-glycyl]GSH[22] as an internal standard. The method is sensitive enough to determine tracer enrichments of [^13^C-cysteine]-GSH with a quantitation limit in the range of 0.3–0.5 mol% excess. The enrichment of [1-^13^C]-cysteine in plasma was also measured as described earlier.[21]

For determination of GSH and cysteine enrichments in the organs, about 0.3–0.5 g tissue samples were homogenized with 1 ml ice-cold DTT (in 1 M acetic acid) solution. The exact wet weight of the tissue was measured by the difference in tube weight before and after addition of the sample. To exactly 500 ml of homogenate, 20 ml of the labeled GSH internal standard was added and the mixed sample was passed through an ion-exchange column. The fraction of the elute containing GSH was collected. GSH concentrations and enrichments were measured, as for whole blood. The enrichment in the column eluate of free cysteine was also determined, as for plasma.

### Calculations

The plasma cysteine flux (Q_cys_) was calculated using the conventional steady-state equation.[21] Absence of a statistically significant slope of plasma cysteine enrichment during the period from 120 to 360 min was confirmed by linear regression analysis.

#### Whole-blood GSH kinetics

The rationale of using stable isotope labeled precursor substrate (in the present case, ^13^C-labeled cysteine) to evaluate product (in the present case, GSH) synthesis rate is based on the steadily increased isotopic enrichment of the product against time. The slope of the product enrichment reflects how fast the precursor incorporates into the product, when the precursor enrichment is in a steady state . Thus, the fractional synthesis rate of whole-blood GSH (FSR_wb,gsh_/day) was calculated from the linear enrichment slope of the labeled [1-^13^C]-cysteine-GSH in the whole blood [K; (molar ratio excess)/min] and the plasma precursor enrichment (Ep; molar ratio excess) of [1-^13^C]-cysteine. This general approach has been described by Jahoor *et al.*[23]

Thus,(1)

The absolute rate of GSH synthesis in the whole blood (ASR_wb;gsh_;μmol/l/day) was calculated from the whole-blood GSH concentration (Cgsh; μmol/l) and the FSR_wb,gsh_

Thus,(2)

#### Organ GSH synthesis rate

The fractional synthesis rate (FSR) of GSH in different organs was calculated from the enrichment of L-[1-^13^C]-cysteine-GSH and that of free L-[1-^13^C]-cysteine in each organ obtained at the end of the tracer infusion. This approach assumes that the incorporation of L-[1-^13^C]-cysteine into the GSH pool in tissues follows single exponential kinetics as previously applied.[23,24] Thus,(3)

where Et is the enrichment of [1-^13^C]-cysteine-GSH at time t (6 h), Eo is the enrichment of precursor [1-^13^C]-cysteine in the organ free amino acid pool, and k is the fractional rate of GSH synthesis. Hence, by algebraic conversion, the equation can be expressed as:(4)

The absolute synthesis rate of GSH in the organ (ASR, in μmol/g wet tissue/day) was calculated from k × GSH concentration in each organ (expressed as μmol/g wet tissue).

### Data presentation and statistics

The results are expressed as means ± standard error of mean (SEM). Statistical evaluation of the data was performed using PROSTAT software (Poly Software International, Inc., Pearl River, NY). All data were examined for normalcy of distribution before carrying out further comparison tests. Independent-sample *t*-test was used to compare the metabolic measurements between the sham burn and burn groups. For those data which did not follow a normal distribution, non-parametric Wilcoxon Ranking Test was used for comparison between these two groups. The probability level below 0.05 was considered significant.

## Results

### Body weight and cysteine kinetics

The body weights for the burn and sham burn animals are shown in Table 2. Burned animals lost about 0.4 kg during the 3 days following burn injury. Before burn injury, the animals food intake was (mean ± SEM) 43.0±3.0 g/kg/ day, which provided 90 kcal/kg/day and 7.1 g protein/kg/ day, including 78 mg methionine (522 μmol)/kg/day and 82 mg cystine (341 μmol)/kg/day. On the first post-burn day, food intake was 35.0 ± 2.1 g/kg/day, but by day 2 it was 45.3 ± 3.2 g/kg/day.

**Table 2:** Whole-blood GSH and plasma cysteine kinetics in sham burn and burned rabbits

	Sham	burn Burned
Body weight (kg)	2.7 ± 0.1	2.5 ± 0.1
Cysteine flux (μmol/kg/h)	29.3 ± 5.1	65.6 ± 8.9^†^
Increment of ^13^C-cysteine-GSH enrichment (molar ratio excess/min)	2.09 × 10^−4^	1.15 × 10^−4†^
FSR_wb,gsh_ (per day)	0.46 ± 0.04	0.38 ± 0.02^¥^
Blood GSH concentration (μmol/l)	1486.0 ± 118.6	1263.8 ± 78.7^§^
Blood GSH synthesis rate (ASR_wb,gsh,_ μmol/min/l)	666.7 ± 58.2	486.9 ± 41.6^¶^

*Data are presented as Mean±SEM, sham burn *n*=7, burned *n*=6

^†^Significantly different from sham burn animals, *P*<0.001 by unpaired *t*-test (equal variance)

^¥^Significantly different from sham burn animals, *P*<0.05 by unpaired *t*-test by Wilcox ranking test

^§^Significantly different from sham burn animals, *P*=0.054 by unpaired *t*-test (equal variance)

^¶^Significantly different from sham burn animals, *P*<0.01 by Wilcox ranking test

**Figure 2:**
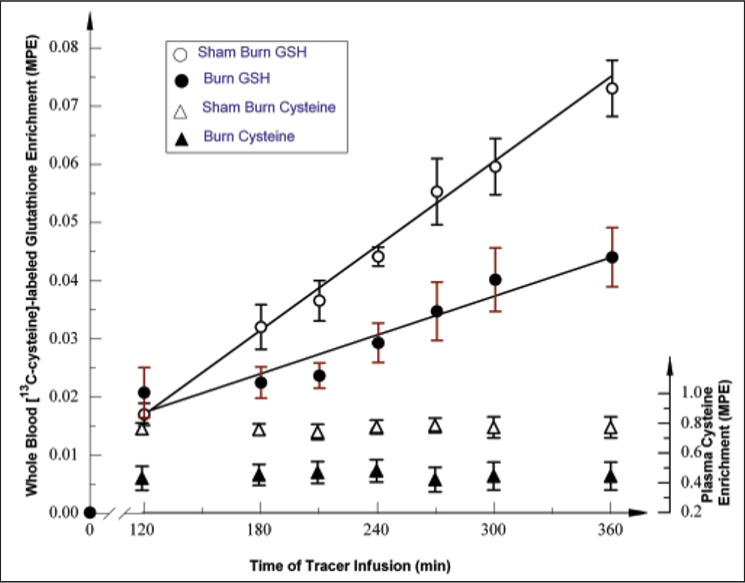
The plasma enrichment (molar ratio excess) of [^13^C]-cysteine and whole-blood [^13^C]-cysteine-GSH in sham burn (*n* = 6) and burned (*n*= 7) rabbits during the 360-min tracer infusion study.

During the last 4 h of tracer infusion studies, plasma cysteine enrichment reached a plateau level, as shown in Figure 2. The plasma cysteine flux was about 220% higher in burned as compared with sham burn animals [Table 2], reflecting, in part, a significant increase in whole-body protein turnover and release of cysteine via proteolysis.[20]

### Blood GSH kinetics

The enrichment of labeled GSH in whole blood showed a progressive increment during the tracer infusion period [Figure 2]. The calculated whole-blood FSR_wb,gsh_ in the burned animals was (mean ± SEM; per day) 0.38 ± 0.02 and significantly lower than for the sham burn controls (*P* < 0.05) [Table 2]. The blood GSH concentration showed a tendency to decline in the burned animals, (*P* = 0.054 by unpaired *t*-test). The absolute rate of whole-blood GSH synthesis (ASR_wb,gsh_) was significantly reduced (*P* < 0.01) by about 30% in burned animals.

### GSH kinetics and concentration in organs

**Figure 3:**
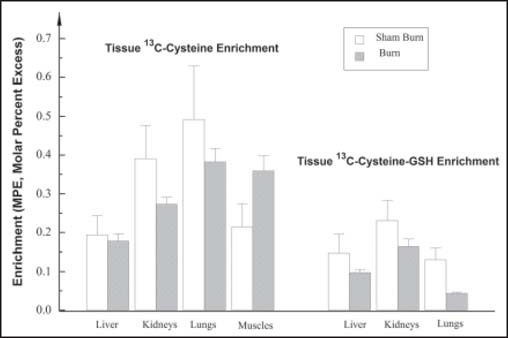
The enrichments (molar ratio excess) of [^13^C]-cysteine and [^13^C]-cysteine-GSH in different tissues in sham burn and burned rabbits after 360 min of tracer infusion.

The enrichments of L-[1-^13^C]-cysteine-GSH and L-[1-^13^C]-cysteine in the liver, lung, and kidney of the two groups of animals are shown in Figure 3. The concentrations, FSR, and ASR of GSH in the organs are summarized in Table 3. Burn injury caused significant reduction in the concentration of GSH in the liver and lungs; the concentration was unaffected in the kidneys. The FSR in the liver (*P* = 0.058) and lungs (*P* < 0.01) decreased after burn injury, but it did not change in the kidneys. As a consequence, the estimated ASR of GSH in the liver (*P* < 0.01) and lungs (*P* < 0.05) showed significant decline with burn injury. The ASR for kidney GSH remained unchanged. We failed to determine a reliable GSH concentration and enrichment in skeletal muscle from burned animals. However, determinations were possible on muscle from sham burn animals, suggesting a significant burn-induced depletion of GSH. Specifically, the GSH concentration in the left quadriceps femoris muscle of sham burn animals was 1.00± 0.37 μmol/g wet weight; FSR was 2.00 ± 0.66/day and the ASR was 2.5 ± 1.2 μmol/day/g wet weight.

**Table 3:** GSH concentration, FSR, and synthesis rates in tissues after burn injury

	GSH concentration (μmol/g wet tissue)		GSH FSR (per day)		GSH synthesis rate (μmol/g wet tissue/day)	
	Sham burn	Burned	Sham burn	Burned	Sham burn	Burned
Liver	2.00±0.17	0.61±0.05*	5.86±1.36	3.76±0.76^§^	10.85±1.15	2.12±0.30^¥^
Lungs	1.15±0.11	0.79±0.08*	1.54±0.40	0.51±0.06*	1.65±0.32	0.40±0.05*
Kidneys	0.20±0.06	0.25±0.04	3.58±0.23	4.06±0.84	0.69±0.18	0.87±0.12

*Significantly different from the sham burn group, *P*<0.05, by Wilcox ranking test

^¥^Significantly different from the sham burn group, *P*<0.01, by Wilcox ranking test

^§^*P*=0.058, by Wilcox Ranking test

## Discussion

Extensive studies reported that GSH concentrations in the blood and tissues are decreased in multiple diseased conditions,[7–10] especially in burn and other critical illness.[15–18] From a dynamic point of view, the reduced GSH level could be related to a negative balance between the changes of GSH synthesis and GSH utilization. The present study can be considered as the first report on the changed dynamics of GSH metabolism instead of merely concentrations in multiple tissues/organs in response to burn injury. Based on the results of the present study, it can be decided what further mechanistic investigations are required.

Using a stable isotope tracer approach and gas chromatography/mass spectrometry technique, we have been able to determine the rate of GSH synthesis in whole blood, lungs, liver, and kidneys in sham burn and burned rabbits. Our findings reveal that the reduction of GSH content in the lungs, liver, muscle, and whole blood, 3 days after a thermal injury, is associated with a significant reduction in the absolute rate of GSH synthesis in the liver and lungs. Since dietary GSH (up to 100 mg/day) is taken up by enterocytes and protects against absorption of peroxidized lipids with minimal release into the blood,[25–27] body GSH is virtually totally synthesized *de novo* in the tissues. Therefore, it can be concluded that a reduced GSH *de novo* synthesis rate accounts for, at least in part, the GSH depletion in different organs following burn injury.

Changes in GSH status in response to severe stressed conditions are time dependent[16] and related to the nature of the stressor.[28] Ikegami[18] and Keller[29] reported decreased tissue GSH concentrations in rat liver during the first few hours of an infection and during septic shock. Hunter and Grimble[30] reported increased concentrations of GSH in the liver, 24 h after stimulation of tumor necrosis factor or endotoxin. Malmezat *et al.*,[31,32] reported increased GSH concentrations in the liver, spleen, skeletal muscle, lung, and heart, but a decrease in whole blood, 2 days after the rats received an injection of live *Escherichia coli*, associated with elevated activities of liver g-glutamylcysteine synthetase and GSH reductase. In humans, at 24–72 h following surgical trauma, there is a decreased concentration of GSH in the skeletal muscle and reduced GSH synthetase capacity. Reid *et al.*[33] also reported reduced concentrations of erythrocyte GSH and lowered FSR and ASR in malnourished children at the time of infection and after the infections had cleared and the edema subsided. The present findings of GSH metabolism after burn injury in an animal model are in agreement with the above reports in surgical patients. Our results further indicated that the reduced GSH concentration and synthesis rates in the liver and lungs occurred at the hypermetabolic and catabolic state of burn injury.[20]

We have previously drawn some general parallels between the current burn-rabbit model and the findings in severely burned patients.[20] Thus, the reduced rate of whole-blood GSH synthesis in burned patients[19] may qualitatively reflect changes in GSH synthesis in organs such as the liver and lungs, which we are unable to probe in humans with our current tracer techniques. Since the whole-blood GSH mostly reflects GSH in red blood cells,[34] the results of the present study are in general agreement with the other reports stating that erythrocytes’ GSH synthesis reflects that in organs, especially the liver.[35]

*De novo* GSH synthesis is mainly regulated by three factors: 1) the intracellular level and activities of glutamate-cysteine ligase (GCL), which catalyzes the rate-limiting first step of GSH synthesis in the γ-glutamyl cycle;[36,37] 2) availability of cysteine and other amino acid precursors, glutamate and glycine; and 3) feedback inhibition of GSH.[28]

GCL consists of a larger catalytic subunit and a smaller modulator subunit. Genes for the subunit proteins are located on separate chromosomes. The expression of GCL is regulated by multiple factors. Under the conditions of severe burn injury and sepsis, impaired insulin function could affect liver GCL expression, as has been demonstrated in type II diabetes condition. The signals involve Phosphoinositide 3-kinase/Protein Kinase B/ p70S6 kinase (PI3K/Akt/p70S6K) pathway[38,39] the induced transforming growth factor (TGF)-β1 after burn injury[40] may also inhibit the transcriptional activity of GCL subunit. Furthermore, GCL subunit can be cleaved by a caspase 3-dependent mechanism under increased apoptosis, which is seen after burn injury[41] Based on the results of the present study, further mechanistic studies will be directed toward the investigations on the molecular basis for reduced GSH synthesis in the g-glutamyl cycle pathways under the burn injury-induced severe inflammatory state.

In addition to enzyme activities, substrate availability may be a more important factor causing the reduction of GSH synthesis in prolonged bed-resting burn patients. Since the clinically used parenteral nutrition solution lacks cysteine and the altered methionine-homocysteine cycle after burn injury may compromise endogenous cysteine synthesis,[42] these factors lead to a reduction of cysteine availability, which contributes to reduced GSH synthesis in burn patients. Conversely, an increased cysteine supply has been shown to improve GSH status in other disease conditions.[7] Extensive literature also suggested that glutamine deficiency is related to reduced GSH synthesis in ICU patients,[43] although the most recent clinical trials questioned the beneficial effect of glutamine supplementation at 30 g/day in these patients.[44]

The present study revealed that the plasma cysteine flux or turnover rate increased by about 36 μmol/kg/h, 3 days after burn injury. This change appears to be more dramatic than the increase in plasma cysteine flux reported by Malmezat *et al.*[32] In rats, 2 days after an *E. coli* challenge. The increased metabolic flux of cysteine is contributed by the following factors: 1) increased proteolysis in the hypermetabolic state of burn injury, which accounts for 61% of the measured increment of 36 μmol/kg/h [Table 2] and 2) increased GSH catabolism, which is supported by the finding that in human subjects, the breakdown of GSH (by the ectoenzyme g-glutamyl transpeptidase) makes a quantitatively important contribution to the plasma metabolic flux of cysteine.[45] Malmezat *et al.*[32] have also suggested that the increased cysteine turnover in infected rats is due, in part, to increased GSH turnover.

In summary, the present study has revealed a significantly reduced rate of GSH synthesis in whole blood, liver, and lungs following burn injury, which accounts for the reduced GSH levels in these tissues, rendering the tissues vulnerable to oxidative damage and impaired mitochondrial function.[46] The reduced GSH synthesis is largely related to substrate availability. GSH concentration and synthesis in the kidney appears to be unaffected, which is related to the fact that kidneys receive GSH by inward transport from circulation.[47] The present study directs further investigations on the mechanism of this reduced GSH availability in organs/tissues, with emphasis on 1) the molecular mechanism of the enzymatic changes in γ-glutamyl cycle, the machinery of GSH synthesis; 2) the mechanism of oxidative damage under the reduced GSH content in tissues; and 3) the interventions that sustain or stimulate GSH synthesis, using organ-directed, nutritional, and/or pharmacological approaches, with the ultimate goal of ameliorating mortality and morbidity of severely burned patients.

## Abbreviations

ASR: Absolute synthesis rate
DTT: Dithiothreitol
FSR: Fractional synthesis rate
GSH: Glutathione
Q_cys_: Plasma cysteine flux
SIM: Selective ion monitoring
WB: Whole blood
